# Lower serum Klotho level and higher systemic immune-inflammation index: an inverse correlation

**DOI:** 10.1186/s12877-023-04349-4

**Published:** 2023-10-11

**Authors:** Ping Chen, Yafen Tang, Liang Luo, Haotian Chen, Xingkang He

**Affiliations:** 1 Nursing Department, Sir Run Run Shaw Hospital, Zhejiang University School of Medicine, Hangzhou, 310016 China; 2 Department of Gastroenterology, Sir Run Run Shaw Hospital, Zhejiang University Medical School, Hangzhou, 310016 China

**Keywords:** SII, Klotho, NHANES, Inflammation

## Abstract

**Objectives:**

Klotho, an anti-aging protein, has been identified to control tissue inflammatory responses. The objective of this research is to determine the linkage between soluble Klotho (S-Klotho) level and systemic immune-inflammation index (SII).

**Methods:**

Eligible participants with complete information of S-Klotho level and SII were selected from the National Health and Nutrition Examination Surveys (NHANES). Subsequently, weighted multivariate linear regression and subgroup analysis were carried out to evaluate the association.

**Results:**

Totally, 11,108 adults with complete data on S-Klotho level, SII and other important covariates were included in final analysis. Multivariate liner regression revealed that high level of S-Klotho was associated with low level of SII after multivariate adjustments (β=-0.08, 95%CI:-0.10- -0.05, P < 0.01). When classifying S-Klotho into tertiles, participants in S-Klotho tertile 3 (Q3) showed a decrease in SII level compared with those in the lowest tertile (Q1) (β=-45.44, 95%CI:-64.41- -26.47, P < 0.01 ). The negative associations remained significant regardless of age and gender, and varied depending on smoking status and BMI subgroups.

**Conclusion:**

S-Klotho level was negatively related to SII after controlling for covariates. Further studies need to validate current findings and explore the fundamental mechanisms.

## Introduction

The scientific community was first made aware of the Klotho gene by Makoto Kuro-o et al. in 1997, when they discovered that Klotho-deficient mice exhibited reduced lifespans and various aging-related phenotypic changes [1]. This led to the suggestion that Klotho may possess anti-aging properties [2]. The alpha-Klotho gene is responsible for encoding a transmembrane protein that is predominantly found in renal tubules. This protein is known as alpha-Klotho and its extracellular domain can be shed to form a soluble variant known as S-Klotho [3]. Studies have shown that S-Klotho has the ability to protect against a range of systemic diseases, such as chronic kidney disease, interstitial lung disease, and cardiovascular events [4–7]. Furthermore, investigations have demonstrated that S-Klotho plays a pivotal role in modulating oxidative stress, apoptosis, cellular senescence, and endothelial function, positioning it as a promising target in the development of treatments for aging-related diseases [8, 9].

There is a growing body of evidence that suggests a relationship between S-Klotho levels and inflammation. Reduced levels of S-Klotho have been associated with a heightened risk of chronic inflammation, whereas increased levels of Klotho have been shown to possess anti-inflammatory effects [10]. For instance, it has been demonstrated through various studies that a low concentration of Klotho is associated with an augmented likelihood of atherosclerosis, which is a pathological state characterized by chronic inflammation of the vascular system [11]. Additionally, higher Klotho levels have been linked to improved function of the immune system and reduced oxidative stress, both of which play pivotal roles in the development of inflammation [12]. It is noteworthy that the association between S-Klotho levels and inflammation is multifaceted and not entirely elucidated. Further investigation is essential to fully comprehend the mechanisms by which Klotho regulates inflammation and its potential as a therapeutic intervention.

The Systemic Immune-Inflammation Index (SII) is a measure obtained by dividing the product of neutrophil counts (N) and platelet counts (P) by lymphocyte counts (L) [13]. This index has been associated with poor prognosis of specific cancer types, such as hepatocellular carcinoma, renal cell carcinoma, colorectal cancer and metastatic castration-resistant prostate cancer [13–15]. It has been established through research that SII can offer an unbiased portrayal of the interplay between the inflammatory response and immunological reaction in patients suffering from tumors [16]. In addition to cancer, SII has also been closely associated with other diseases, such as autoimmune illnesses, cardiovascular diseases, and dementia [17–20]. Compared with other inflammatory biomarkers, SII provides a more comprehensive evaluation of the systemic immune-inflammatory status by incorporating multiple components (neutrophil counts, platelet counts, lymphocyte counts). It has been suggested that higher Klotho levels are associated with lower levels of several inflammatory markers. A study conducted by Polat et al. demonstrated an inverse correlation between klotho level and elevated C-reactive protein level in elderly individuals aged 65 and above [21]. A further study conducted on a broader population revealed that serum Klotho level was inversely associated with uric acid, C-reactive protein, and white blood cell count, while positively correlated with mean platelet volume [22]. To date, there have been no evaluations of the relationship between serum Klotho level and SII. The potential influence of S-Klotho levels on SII or vice versa is currently unclear. The objective of the current investigation, which utilizes information obtained from the National Health and Nutrition Examination Survey (NHANES), is to examine and evaluate the correlation between S-Klotho and SII in general population.

## Method

### Study population

**Fig. 1:**
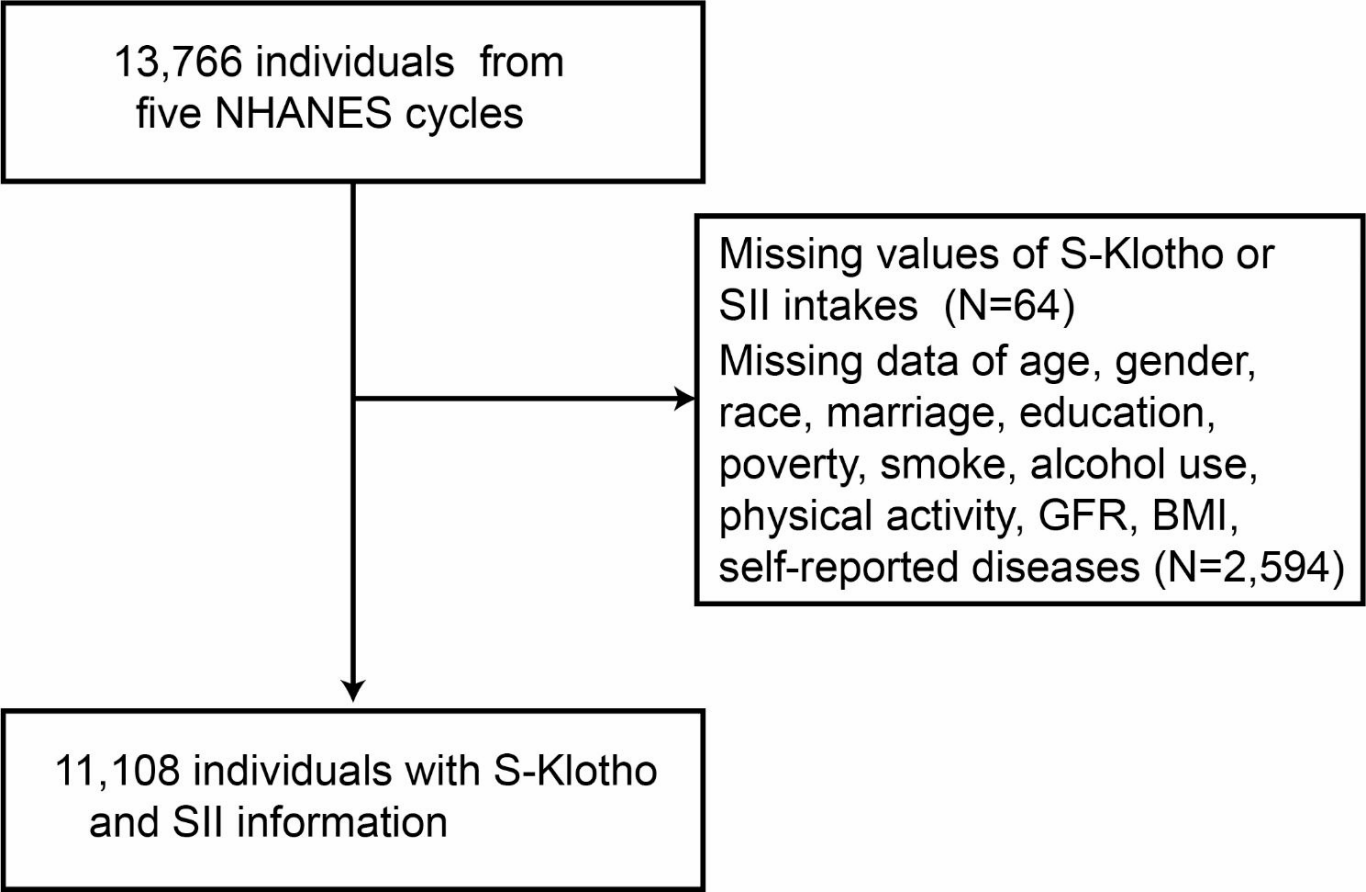
Flowchart for participant selection from NHANES.

NHANES is a significant ongoing initiative of the National Center for Health Statistics (NCHS) at the Centers for Disease Control and Prevention that aims to assess the health and nutritional status of noninstitutionalized residents in the United States. The survey utilizes a complex, stratified, multistage, probability sampling design to select participants that are representative of the target population. Further details about NHANES can be found on their website (https://www.cdc.gov/nchs/nhanes/about_nhanes.htm). Prior to conducting this study, all participants were required to provide written informed consent and the study was granted approval by the NCHS Ethics Review Board. The scope of this research was limited to five consecutive NHANES cycles, specifically those from 2007 to 2008, 2009–2010, 2011–2012, 2013–2014, and 2015–2016, with a total of 13,766 individuals included. In order to be eligible for inclusion in the study, participants needed to have available data pertaining to their age, gender, ethnicity, marital status, education, poverty, smoking status, alcohol drinking status, energy intake, body measurements, waist circumference, medication usage, SII, and Klotho. Any participants with missing or unknown data were excluded from the final analysis. Ultimately, a total of 11,108 individuals met the inclusion criteria and were included in the study (as illustrated in Fig. 1).

### Calculation of SII

According to relevant literature, the Systemic Immune-Inflammation Index (SII) is determined through a formula that involves multiplying the platelet count and neutrophil count, and then dividing the outcome by the lymphocyte count [13]. The measurements for lymphocyte, neutrophil, and platelet counts are expressed as ×10^3^ cells/µl and are typically obtained using automated hematology analysis devices.

### Measurement of S-Klotho levels

In this study, the levels of soluble alpha-Klotho (S-Klotho) were measured in frozen serum specimens obtained from participants aged 40 to 79 years who were part of the National Health and Nutrition Examination Survey (NHANES). To ensure consistency and accuracy, a commercially available ELISA kit (IBL International, Japan) was utilized for the measurement of S-Klotho levels. [23] The instructions provided on the NHANES website were meticulously followed during the entire process. To minimize any potential errors, each sample was analyzed twice, resulting in duplicate measurements for each participant which were then averaged. The resulting data was then categorized into three groups (Q1, Q2, and Q3) based on the distribution of S-Klotho levels, enabling the researchers to compare and analyze the levels of S-Klotho within each group.

### Assessment of covariates

To evaluate the potential impact of factors that could affect the results, we have identified a number of variables that may act as confounding factors. These variables include age (measured in years), gender (male or female), ethnicity (categorized as non-Hispanic white, non-Hispanic black, Mexican American, or other Hispanic), marital status (unmarried or married), education level (categorized as grade or less, high school, or college or more), poverty level (measured as the family income-to-poverty ratio), smoking status (categorized as never, former, or current smoker), alcohol consumption (categorized as never, former, mild, moderate, or heavy), healthy eating index-2015, total energy intake (measured in calories), body mass index (BMI), use of medication, glomerular filtration rate, physical activity (categorized as yes or no), and self-reported chronic diseases such as hypertension, diabetes, stroke, heart attack, congestive heart failure (CHF), coronary heart disease (CHD), and cancer. Physical activity was divided into two categories based on whether individuals met the recommended level of physical activity (150 min or more of moderate-to-vigorous physical activity per week) or not (less than 150 min of moderate-to-vigorous physical activity per week) [24].

### Statistical analysis

In accordance with NHANES guidelines, weighted analyses were conducted on a complex sampling survey. In accordance with the analytical protocol of NHANES, we utilized WTMEC2YR as the sample weight and determined the ten-year sample weight to be 1/5 of WTMEC2YR for our analysis (https://wwwn.cdc.gov/nchs/nhanes/analyticguidelines.aspx). The basic characteristics of included participants were presented as mean ± standard deviation (SD) for continuous variables and percentage for categorical variables. To compare baseline characteristics across various S-Klotho levels, weighted one-way analysis of variance (ANOVA) and chi-square test were employed for continuous variables and categorical variables respectively. Furthermore, we conducted weighted multivariate linear regression analysis to assess the relationship between S-Klotho and SII in various models. Model 1 did not adjust for confounding variables, while Model 2 adjusted for age, and Model 3 adjusted for age, survey cycle, gender, ethnicity, marital status, education, poverty, eGFR, body mass index, energy intake, healthy eating index, smoke status, alcohol drinking status, physical activity, and self-reported chronic diseases, including hypertension, diabetes, stroke, heart attack, congestive heart failure, coronary heart disease, and cancer. To investigate the relationship between S-Klotho and SII in different subgroups, we carried out subgroup analysis, stratifying by age (≤ 60/>60 years), gender (male/female), smoking status (never/former/now), and BMI (normal weight/overweight/obesity). The coefficient and P-value of subgroup analysis were calculated by weighted multivariate linear regression (adjusted for age, plus survey cycle, gender, ethnicity, marital status, education, poverty, eGFR, body mass index, energy intake, healthy eating index, smoke status, alcohol drinking status, physical activity, and self-reported chronic diseases). We conducted a subgroup analysis using S-Klotho as the categorical variable, with β representing the coefficient of the highest tertile of S-Klotho level (Q3) compared to the lowest tertile (Q1). We used the “nhanesR” package to extract and analyze data. The R software (version 4.2) was utilized for all analyses, and all statistical tests were conducted as two-sided. The threshold for statistical significance was set at P < 0.05.

## Results

The final analysis of the current study incorporated a total of 11,108 participants who satisfied the predetermined inclusion and exclusion criteria. The baseline weighted characteristics of the included population stratified by the S-Klotho tertiles were presented in the Table 1. Significant variations were noted in age, gender, ethnicity, smoking and drinking habits, healthy eating index, eGFR, SII, medication use, and history of hypertension, diabetes, CHF, CHD, and cancer among the S-Klotho tertiles. However, no significant differences were observed in marital status, education status, poverty, BMI, physical activity, energy intake, and history of stroke or heart attack.

**Table 1:** Characteristics of included population in the NHANES based on S-Klotho tertiles (N = 11,108)

Characteristic	S-Klotho Tertiles			P value
	Q1	Q2	Q3	
Age(years)	57.16 ± 10.67	56.11 ± 10.10	55.27 ± 10.12	**< 0.01**
Gender				**< 0.01**
Female (%)	32.23	33.69	34.08	
Male (%)	35.14	35.27	29.59	
Ethnicity				**< 0.01**
Non-Hispanic white (%)	34.36	35.1	30.53	
Mexican American (%)	31.76	36.43	31.81	
Non-Hispanic black (%)	31.34	27.72	40.94	
Other Hispanic (%)	29.05	32.64	38.31	
Other (%)	32.69	35.41	31.89	
Marital status				0.53
Unmarried (%)	33.06	34.14	32.8	
Married (%)	33.92	34.61	31.47	
Education				0.1
Grade or less (%)	35.57	32.44	31.99	
High school (%)	35.37	35.11	29.52	
College or more (%)	32.54	34.71	32.75	
Family income-to-poverty ratio	3.26 ± 1.60	3.33 ± 1.60	3.29 ± 1.64	0.35
Smoking				**< 0.01**
Never (%)	30.99	34.7	34.31	
Former (%)	36.03	34.04	29.92	
Now (%)	37.11	34.45	28.44	
Alcohol drinking				**< 0.01**
Never (%)	28.4	36.38	35.22	
Former (%)	33.85	32.63	33.52	
Mild (%)	31.52	34.08	34.4	
Moderate (%)	36.78	33.35	29.87	
Heavy (%)	38.95	37.57	23.48	
Energy intake (kcal/day)	2093.13 ± 884.65	2132.32 ± 945.49	2084.06 ± 872.19	0.2
Healthy Eating Index-2015	51.66 ± 13.55	52.41 ± 13.71	52.58 ± 14.12	**0.04**
Glomerular filtration rate (ml/min/1.73m^2^)	83.75±	86.80±	89.02±	**< 0.01**
Body Mass Index (kg/ m^2^)	29.65 ± 6.19	29.78 ± 6.76	29.39 ± 6.86	0.15
Klotho (pg/ml)	580.31 ± 92.63	802.76 ± 60.93	1164.81 ± 279.97	**< 0.01**
Systemic immune-inflammation index	570.38 ± 324.83	551.37 ± 385.65	517.01 ± 310.31	**< 0.01**
Physical activity				0.14
No (%)	33.72	32.68	33.6	
Yes (%)	33.6	35.02	31.38	
Use of medication (%)				**< 0.01**
Yes (%)	34.84	34.65	30.51	
No (%)	30.7	33.97	35.33	
Self-reported chronic diseases				
Hypertension (%)	35.71	33.12	31.18	**0.03**
Diabetes (%)	37.02	31.72	31.26	**0.04**
Stroke (%)	40.78	29.36	29.86	0.05
Heart attack (%)	36.41	35.54	28.05	0.25
CHF (%)	42.82	33.14	24.03	**< 0.01**
CHD (%)	41.86	31.59	26.56	**0.01**
Cancer (%)	35.52	37.56	26.92	**< 0.01**

Data are expressed as mean ± standard deviation (SD) for continuous variables and as percentages (%) for categorical variables. The P value is calculated by weighted one-way analysis of variance (ANOVA) for continuous variables and weighted chi-square test for categorical variables. Abbreviations: CHF, Congestive heart failure; CHD, Coronary heart disease, NHANES, National Health and Nutrition Examination Survey

When analyzed as a continuous variable, higher levels of S-Klotho were observed to be inversely correlated with lower levels of SII. Upon conducting minimal and full adjustments, a one-unit increase in S-Klotho was found to be significantly associated with a reduction of 0.09 and 0.08 in SII, respectively, in models I and II (Table 2). Furthermore, this relationship persisted even when S-Klotho was categorized into three groups (Q1, Q2, and Q3). Following the multivariate adjustment that controlled for variables such as age, survey cycle, gender, ethnicity, marital status, education, poverty, eGFR, body mass index, energy intake, healthy eating index, smoke status, alcohol drinking status, physical activity, and self-reported chronic diseases, it was observed that participants exhibiting the highest S-Klotho level demonstrated a 45.44 reduction in SII when compared to those with the lowest level (Table 2). Participants in the second tertile of S-Klotho level did not show a significant difference in SII when compared to those in the lowest tertile, as indicated in Table 2. To further assess the stability of the negative associations, we conducted subgroup analysis. Stratified analyses by age and gender demonstrated significant associations between S-klotho and SII in all subgroups (Table 3). Stratified by smoking status, significant associations were observed in participants who had never smoked or were former smokers, rather than in those who currently smoked (Table 3). Stratified by BMI, a negative association between S-klotho and SII was only observed in the subgroup of participants with obesity, whereas no significant association was found in the subgroups with normal weight or overweight (Table 3).

**Table 2:** Weighted multivariate liner regression analysis of association between S-Klotho level and Systemic immune-inflammation index

S-Klotho level	Crude Model β (95%CI)	P	Model I β (95%CI)	P	Model II β (95%CI)	P
**Continuous variable**	-0.09 (-0.11, -0.07)	**< 0.01**	-0.09 (-0.11, -0.07)	**< 0.01**	-0.08 (-0.10, -0.05)	**< 0.01**
**Categorical variable**						
**Q1**	Reference		Reference		Reference	
**Q2**	-19.01 (-39.44, 1.42)	0.07	-18.8 (-39.25, 1.65)	0.07	-18.08 (-38.03, 1.87)	0.07
**Q3**	-53.37 (-72.82, -33.92)	**< 0.01**	-52.99 (-72.47, -33.51)	**< 0.01**	-45.44 (-64.41, -26.47)	**< 0.01**
**P for trend**		**< 0.01**		**< 0.01**		**< 0.01**

Weighted linear regression analysis is used to calculate the β and P-value in the Crude model, while the β and P-value in the models I and II are obtained using weighted multivariate linear regression. Crude model adjusted for nothing, Model I adjusted for age; Model II adjusted for age, plus survey cycle, gender, ethnicity, marital status, education, poverty, eGFR, body mass index, energy intake, healthy eating index, smoke status, alcohol drinking status, physical activity, and self-reported chronic diseases, including hypertension, diabetes, stroke, heart attack, congestive heart failure, coronary heart disease, cancer

**Table 3:** Stratified analyses of association between S-Klotho level (the lowest tertile Q1 as reference) and systemic immune-inflammation index

Variables	Model II β (95%CI)	P-value
**Stratified by Age**		
≤ 60 years	-37.21(-63.22, -11.20)	**0.01**
>60 years	-57.4(-88.04, -26.76)	**< 0.001**
**Stratified by Gender**		
Female	-57.32(-83.17, -31.48)	**< 0.0001**
Male	-31.98(-56.60, -7.35)	**0.01**
**Stratified by Smoke**		
Never	-50.89( -75.02, -26.77)	**< 0.0001**
Former	-43.48(-80.28, -6.68)	**0.02**
Now	-31.53(-87.06, 24.01)	0.26
**Stratified by body mass index**		
Normal weight	-32.66( -78.03, 12.72)	0.15
Overweight	-20.8( -53.20, 11.59)	0.20
Obesity	-77.66(-103.93, -51.40)	**< 0.0001**

Model II adjusted for age, plus survey cycle, gender, ethnicity, marital status, education, poverty, eGFR, body mass index, energy intake, healthy eating index, smoke status, alcohol drinking status, physical activity, and self-reported chronic diseases, including hypertension, diabetes, stroke, heart attack, congestive heart failure, coronary heart disease, cancer. The strata variable was not taken into account when stratifying by itself. The β and P-value are calculated by weighted multivariate linear regression and β refer to the coefficient in the highest tertile of S-Klotho level (Q3) to those in the lowest tertile (Q1)

## Discussion

In current study, we revealed a noteworthy negative correlation between S-Klotho concentration and SII, even after controlling for multiple confounding factors. Further subgroup analysis had been established that this negative association remained significant regardless of the age and gender. Some heterogeneity could be observed between different smoking status and BMI subgroups. Further research is necessary to validate our discoveries and investigate the particular mechanisms that are causing these observed disparities. The findings of this study contributed to the growing body of evidence supporting the involvement of S-Klotho in immune and inflammatory processes [10].

The concept of the Systemic Immune-Inflammation Index (SII) [13]was introduced as a means of quantifying the interplay between systemic inflammation and the body’s immune response. This metric was derived from the counts of neutrophils, lymphocytes, and platelets in peripheral blood, serving as a comprehensive gauge of the balance between the two aforementioned physiological processes. Prognostic value of SII in various types of cancers had been indicated by previous meta-analysis, such as gastric cancer, pancreatic cancer, urinary system cancers. [25–27] Recent studies had proposed that the systemic immune-inflammation index (SII) could serve as a precise evaluation tool for measuring levels of inflammation. Studies had indicated that increased SII levels were linked to a greater probability of increased disease activity in peripheral arterial disease, ulcerative colitis, osteoporosis in postmenopausal women, and depression in patients with diabetes mellitus [28–31]. Compared with inflammatory biomarkers, such as IL10, TNFα/IL10 ratio, CRP, and uric acid, the components measured in SII are derived from peripheral blood counts, making them more convenient and readily available in clinical practice, which is an advantage of using SII. Furthermore, when comparing SII to markers such as white blood cell count and mean platelet volume, SII had provided additional information. While white blood cell count and mean platelet volume reflect specific cellular components involved in the immune response and thrombotic processes, SII incorporates multiple blood cell types, including lymphocytes, neutrophils, and platelets, taking into account a broader spectrum of immune and inflammatory activity, potentially providing a more comprehensive assessment of the systemic immune-inflammation status.

Increasing evidence is demonstrating S-Klotho might have a protective influence in a variety of disorders, particularly in inflammatory states. A number of research studies had indicated a connection between S-Klotho and inflammatory cytokines. Martín-Núñez and colleagues conducted a case-control study on individuals with a history of cardiovascular disease. They found that decreased levels of S-Klotho were significantly correlated with a pro-inflammatory state characterized by reduced levels of IL10, an increased TNFα/IL10 ratio, and elevated levels of CRP. [32] The findings of Wu et al. were consistent in demonstrating that S-Klotho was inversely associated with well-established inflammatory biomarkers, such as uric acid, C-reactive protein, white blood cell count, and mean platelet volume [22]. Despite numerous studies mentioning the connection between S-Klotho and inflammatory cytokines, the correlation between S-Klotho and SII had yet to be fully understood. In the present study, we had established for the first time that the level of S-Klotho is inversely and significantly correlated with SII, and this negative correlation persists regardless of the age and gender of the subjects. Nevertheless, it was observed that there was some heterogeneity and the correlation between S-Klotho level and SII varied among different groups according to smoking status and BMI. Our results were consistent with the notion that S-Klotho could be a reliable marker of systemic inflammation, as previously suggested by aforementioned studies. Due to cross-sectional design of the current study, no causality between S-Klotho and SII could be established. It is yet to be determined whether Klotho could serve as an anti-inflammatory agent to reduce chronic inflammation. Further prospective studies should be conducted to examine if S-Klotho could be a bioactive substance that could impede inflammation and thus, reduce chronic inflammation.

While the correlation between S-Klotho and SII has been established, the underlying biological mechanism is not yet fully understood. While the exact function of S-Klotho remains unclear, several research studies have suggested that it might be a crucial factor in the pathophysiology and escalation of inflammation. For example, downregulation of renal Klotho expression had been correlated with amplified inflammation in the kidneys of diabetic mice [33]. According to the study conducted by Zhao et al., Klotho had functioned as a modulator of inflammation by inhibiting the synthesis of inflammatory proteins associated with NF-κB. This process had been facilitated by the phosphorylation of Ser536 in the transactivation domain of RelA through a specific mechanism [34]. Furthermore, Klotho had also been shown to stimulate the production of anti-inflammatory factors. The production of Klotho, which could be induced by calcitriol and transfection with a Klotho gene-containing plasmid, had been found to upregulate the secretion of IL-10 in human monocytes stimulated with lipopolysaccharide, leading to an immunosenescent-like phenotype [35]. Through its influence on Toll-like Receptor 4 (TLR4) and inhibition of the insulin-like growth factor 1 signaling cascade, Klotho was shown to effectively reduce oxidative stress. This activation of FoxO transcription factors resulted in the induction of manganese superoxide dismutase expression and the removal of reactive oxygen species, ultimately leading to a decrease in oxidative stress [36]. The initiation of proinflammatory signaling induced by lipopolysaccharide is commenced through the activation of NF-κB. This leads to an increase in TLR4 levels in macrophages, renal epithelial cells, and inflamed kidneys, while simultaneously causing a decrease in the expression of Klotho. The reciprocal inhibition of Klotho and TLR4 was established [37]. Furthermore, Klotho had the ability to downregulate the production of intercellular adhesion molecule-1 (ICAM-1), which had been a proinflammatory factor induced by TNF-α, IL-1β, and TXNIP. This downregulation of ICAM-1 was mediated by Klotho’s inhibition of TXNIP, resulting in the prevention of leukocyte infiltration into tissues [38].

This study represented a pioneering effort in exploring the correlation between S-Klotho levels and SII in a population-based cohort. Its unique contribution to the field is further strengthened by its large sample size, which is representative of the non-institutionalized population in the USA and has been collected using standardized methods, thus minimizing measurement bias. However, it is important to acknowledge the limitations of this study. Firstly, its retrospective design precludes the establishment of causal relationships. Secondly, the existence of residual confounding may still have an impact on the correlation between S-Klotho and SII despite adjusting for potential confounders. Finally, the generalizability of our findings to other populations, such as the young population, remains uncertain and requires further investigation.

In conclusion, our study found a significant and negative correlation between S-Klotho levels and SII after controlling for various confounding factors. The inverse relationships remained significant in certain subgroups. Further research is required to validate these results in other populations and to clarify the causal link between S-Klotho and SII. Additionally, supplementary investigations are recommended to reveal the underlying fundamental mechanisms that contribute to this relationship.

## Data Availability

This dataset was derived from the National Health and Nutrition Examination Survey (NHANES), which is accessible to the public on the Centers for Disease Control and Prevention (CDC) website (https://www.cdc.gov/nchs/nhanes/) through the internet.

## Abbreviations

SII: Systemic immune-inflammation index
S-Klotho: Soluble Klotho
BMI: Body mass index
NHANES: National Health and Nutrition Examination Surveys
NCHS: National Center for Health Statistics
ELISA: Enzyme-linked immunosorbent assay
